# Validation of the Thai version of the Neurological Disorders Depression Inventory for Epilepsy (NDDI-E): Screening for major depressive disorder in patients with epilepsy

**DOI:** 10.1192/j.eurpsy.2023.1609

**Published:** 2023-07-19

**Authors:** S. Kuladee, T. Prachason, T. Buranapichet, P. Rodwanno, A. Boongird

**Affiliations:** 1 Department of Psychiatry, Faculty of Medicine, Ramathibodi Hospital; 2 Department of Internal Medicine, Faculty of Medicine, Ramathibodi Hospital, Mahidol University, Bangkok, Thailand

## Abstract

**Introduction:**

Depression has been recognized as a common comorbidity in patient with epilepsy and is associated with low quality of life. Regular screening for depression may aid in early detection and enhance quality of life.

**Objectives:**

To validate the Thai version of the Neurological Disorders Depression Inventory for Epilepsy (NDDI-E).

**Methods:**

The English version of NDDI-E was translated into Thai. Patients with epilepsy were enrolled at the outpatient neurology clinic from May 2019 to September 2019. Demographic data and clinical characteristics were collected. Participants underwent a psychiatric structured interview using the Mini-International Neuropsychiatric Interview (M.I.N.I.) as a gold standard for the diagnosis of major depressive disorder. Then, participants completed the NDDI-E. The internal consistency was measured by Cronbach’s alpha coefficient. The validity of the Thai version of the NDDI-E was assessed using the receiver operating characteristic (ROC) curve analysis. Youden’s index was used to determine the optimal cut-off score of the Thai version of the NDDI-E.

**Results:**

A total of 115 patients with epilepsy completed the evaluation. Twenty-three patients (20%) had major depressive disorder according to M.I.N.I. criteria. The Cronbach’s alpha coefficient of the Thai version of the NDDI-E was 0.826. The area under the ROC curve was 0.995. A cut-off score greater than 17 provided a sensitivity of 95.65%, a specificity of 97.83%, a positive predictive value of 91.67% ,and a negative predictive value of 98.90%.

**Table 1:** Demographic and clinical characteristics of the study population.

	Non-depressed (N = 92)	Major depression (N = 23)	*P*-value
Age in years, median (IQR)	31 (22)	28 (30)	0.637
Female, N (%)	50 (54.3)	15 (65.2)	0.347
Comorbid medical illnesses, N (%)			0.009
Present	40 (43.5)	17 (73.9)	
Absent	52 (56.5)	6 (26.1)	
Years since onset of seizures, mean (SD)	19.4 (11.9)	16.4 (14.1)	0.297
Seizure free for the last 6 months, N (%)	55 (59.8)	10 (43.5)	0.158
NDDI-E score, median (IQR)	12 (5)	19 (4)	<0.001

**Table 2:** Corrected item-total correlation and Cronbach’s alpha if an item is deleted from the NDDI-E.

	Corrected item-total correlation	Cronbach’s alpha if item deleted
1. Everything is a struggle	0.554	0.807
2. Nothing I do is right	0.590	0.800
3. Feel guilty	0.573	0.803
4. I’d be better off dead	0.643	0.788
5. Frustrated	0.660	0.784
6. Difficulty finding pleasure	0.548	0.808

**Conclusions:**

The Thai version of the NDDI-E is a valid screening tool for major depressive disorder in patients with epilepsy.

**Disclosure of Interest:**

None Declared

